# Characterization of Chiral Nanostructured Surfaces Made via Colloidal Lithography

**DOI:** 10.3390/nano13152235

**Published:** 2023-08-02

**Authors:** Sabine Portal, Carles Corbella, Oriol Arteaga, Alexander Martin, Trinanjana Mandal, Bart Kahr

**Affiliations:** 1 Department of Mechanical and Aerospace Engineering, George Washington University, Washington, DC 20052, USA; 2 Experimental Physics II, Ruhr-University Bochum, 44780 Bochum, Germany; 3 Department of Applied Physics, University of Barcelona, 08028 Barcelona, Spain; oarteaga@ub.edu; 4 Department of Chemistry and Molecular Design Institute, New York University, New York, NY 10003, USA; atm343@nyu.edu (A.M.); tm1454@nyu.edu (T.M.); bk66@nyu.edu (B.K.)

**Keywords:** colloidal lithography, ion beam etching, nanostructured surfaces, chirality, polarimetry

## Abstract

Optically anisotropic materials were produced via colloidal lithography and characterized using scanning electronic microscopy (SEM), confocal microscopy, and polarimetry. A compact hexagonal array mask composed of silica sub-micron particles was fabricated via the Langmuir–Blodgett self-assembly technique. Subsequently, the mask pattern was transferred onto monocrystalline silicon and commercial glass substrates using ion beam etching in a vacuum. Varying the azimuthal angle while etching at oblique incidence carved screw-like shaped pillars into the substrates, resulting in heterochiral structures depending on the azimuthal angle direction. To enhance the material’s optical properties through plasmon resonance, gold films were deposited onto the pillars. Polarimetric measurements were realized at normal and oblique incidences, showing that the etching directions have a clear influence on the value of the linear birefringence and linear dichroism. The polarimetric properties, especially the chiroptical responses, increased with the increase in the angle of incidence.

## 1. Introduction

Nanotechnology has created opportunities for manipulating and controlling matter at the atomic and molecular scales. Chiral structures, which consist of elements whose mirror images cannot be superimposed, are interesting for applications involving the control of photonic signals. Researchers can design and engineer materials with tailored optical properties by acting on their nanostructures. To this end, understanding the relationship between (1) the surface morphology of anisotropic and/or chiral nanostructures and (2) the polarization of light is a crucial step [1]. Here, we evaluate the optical consequences of building chiral nanoscopic features into a substrate subjected to ion bombardment after partial protection with a colloidal mask.

Anisotropic 2D crystals are in-plane periodic structures showing directional properties. They can be produced either by bottom-up or top-down approaches. From the bottom-up approach, materials can be synthesized by self-assembled molecules or colloids. Self-assembled monolayers are auto-organized arrays of particles often in the form of “2D” crystals [2]. From the top-down approach, lithography can be applied to featureless materials to produce sub-micrometric or nanometric patterns. Notable examples of lithography include the following:

- Electron beam lithography (EBL) [3,4,5];
- Nanoimprint lithography [6];
- Interferometric lithography [7];
- Colloidal lithography [8,9];
- Hole-mask colloidal lithography [10,11,12].

Directional etching via a plasma or ion beam of a surface supporting a colloidal crystalline monolayer is a common strategy in lithography. It can be used to transfer the colloidal pattern onto the underlying substrate as ions sputter both the particles and the exposed regions of the substrate between the particles. Hence, it is a cost-effective process used to etch substrates and transfer structural characteristics inherited from the overlayer [13]. Chiral structures can be created using these methods. Microscopic gammadions were made using a combination of EBL and ion beam milling [4]. Chiral crescent nanostructures, which are active in the near-infrared region, were obtained by sequentially changing the deposition angle of gold on isolated nanoparticles [14]. The extension of optical chirality to the visible spectrum was achieved by designing Au ‘nanohooks’ using hole-mask colloidal lithography [15]. Recently, chiral metamaterials that are tunable within the UV-visible range were fabricated on flexible substrates [16].

The mechanism regulating light polarization in chiral nanostructures is based on absorption/re-emission phenomena, which are effective through a coupling between incident electromagnetic waves and the geometry of the nano-objects. The optical response of such structures can be further enhanced by modifying the chemical composition on the device surface. Over the past decade, chiral plasmonic materials with structural variance well matched to the wavelength of visible light, even better than molecules, have reinvigorated chiroptical spectroscopy [17,18,19]. Surface plasmon polariton (SPP) excitation is extremely sensitive to surface changes, to the permittivity function of the dielectric layer, to the metal surface functionalization, and to bio-chemical reactions on the metal surface. Some surface plasmon resonance (SPR) sensing devices consist of grating-coupled surface plasmon resonance (GCSPR) that uses gratings coated with a thin gold layer. The surface plasmon wavevector, *k*_sp_, matches the component of the light wavevector parallel to the plane of the sample, *k*_//_, and the reciprocal lattice vector of the grating [20]. Some reports [21] have claimed that the detection of chiral molecules can be enhanced by up to 10^6^-fold using metallic chiral nanostructures, thereby enabling the detection of very low quantities of chiral molecules like proteins.

The purpose of this work is to produce materials with optical activity at a large scale by combining both bottom-up and top-down material synthesis. Previously, surface nanopatterning was realized via colloidal lithography on silicon wafers and glass substrates [22]. The fabrication process consisted of dry etching with argon ions, accelerated in vacuum, using colloidal crystals as sacrificial templates; the incidence angle was varied, and the azimuthal angle remained constant. The colloidal crystalline overlayers served as a cost-effective mask for nanostructuring large surface areas. In the present paper, we made 3D structures in three steps by rotating the sample around its azimuthal plane under oblique incidence. Etching proceeded under stepwise clockwise (CW) and counterclockwise (CCW) rotation directions. The fabrication stages encompassed particle production, self-assembly of a colloidal mask, and an etching stage that transferred the pattern of the particle arrangement onto the underlying substrate. We measured the change in the optical activity for the samples fabricated with opposite senses of the azimuthal rotation during etching. Finally, a metal film was deposited onto the nanopatterned surface to assay the role of plasmon coupling in enhancing responses to circularly polarized light.

## 2. Materials and Methods

### 2.1. Material Fabrication

#### 2.1.1. Particle Synthesis

Silica spheres of around 550 nm in size were synthesized via a simple sol-gel process based on the Stöber method [23]. Ethanol and distilled water solutions of NH_4_OH were mixed and a small quantity of tetraethylorthosilicate (TEOS) was added. At a critical concentration of NH_4_OH, the solution became milky as the size of the silica particles became comparable to the wavelength of the visible light. The molar proportions TEOS:NH_4_OH:H_2_O:EtOH were 1:18:9:126 [24]. Finally, aminopropyltriethoxysilane hydrophilic surfactant (APTS) was introduced to the solution, bringing the molar ratio APTS:TEOS to 1, to facilitate the fabrication of the crystal monolayer via the Langmuir–Blodgett technique.

#### 2.1.2. Colloidal Crystal Deposition

The sub-micron particles were diluted in a mixture of methanol and chloroform in a proportion of 1:3 by volume and sonicated for 1 h before the solution was spread on the water surface in a Langmuir–Blodgett trough by dropping solution aliquots from a Hamilton syringe. After this step, the barriers of the trough were brought closer, with a constant rate of 10 mm/min up to a surface pressure of 5 mN m^−1^. Silicon wafers and commercial glass were used as substrates. The substrate was lifted from the trough with a speed of 2 mm per min at constant pressure, dragging a monolayer of silica sub-micron particles on its surface.

#### 2.1.3. Directional Ion Beam Etching

**Figure 1:**
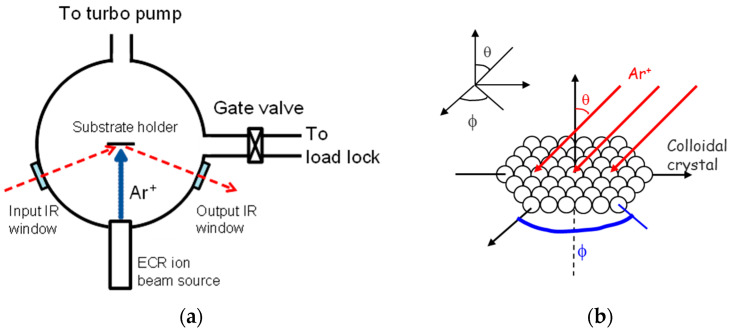
(**a**) Sketch (top view) of the beam chamber equipped with an orientable substrate holder facing an electron cyclotron resonance (ECR) plasma source. The chemical state of the sample was monitored in situ via FTIR. The red dashed arrow represents the path of the IR light beam. Figure adapted from [25]. (**b**) Scheme of a colloidal crystal on a substrate, which is submitted to directional ion beam etching. The etching angles *θ* and *ϕ* are indicated.

Ion beam etching was performed in a vacuum beam reactor (Figure 1) equipped with an electron cyclotron resonance (ECR) plasma source acting as ion gun (TecTra Gen2) [25,26]. The source was set to send argon ions at an energy of 500 eV and an ion flux of about 100 μA/cm^2^, as measured using a Faraday cup. The beam chamber was pumped down to a base pressure lower than 10^−5^ Pa. The samples were introduced to the beam through a load-lock chamber, and they were placed on an electrically grounded holder on an XYZ stage. The holder was 90 mm from the ion gun and was tilted to the desired etching angle, *θ*, and azimuthal angle, *ϕ*. The ion gun was operated at 1 standard cubic centimeter per minute of Ar, resulting in a residual pressure of around 4 × 10^−2^ Pa. The pressure was low enough to maintain the directionality of the ion beam by minimizing scattering.

In situ Fourier-transform infrared spectroscopy (FTIR) characterization was performed using a Bruker IFS 66 FTIR spectrometer. For this analysis, the samples were etched at normal incidence with 500 eV Ar^+^ and an ion flux of 100 μA/cm^2^ was measured using the Faraday cup.

#### 2.1.4. DC Sputtering of Gold Coating

Gold coatings with thicknesses of 10, 24, and 40 nm were deposited on the samples using a sputtering chamber with a rotating sample holder to deposit uniform coatings.

### 2.2. Characterization

#### 2.2.1. SEM and Confocal Microscopy

Prior to the etching, electron microscopy images were captured using a Hitachi H-4100FE field-emission scanning electron microscope (FESEM). Following the etching steps, a Zeiss LEO 1530 Gemini FESEM and Zeiss Merlin FESEM were employed to obtain images. The samples were introduced in the SEM chamber as prepared when the substrate was made of doped silicon. For glass substrates, a metallic connection was made between the sample and the SEM holder to ensure electrical conductivity. The acceleration voltage was fixed to 30 kV for the Hitachi FESEM, 10 kV for the LEO FESEM, and 5 kV for the Merlin FESEM.

Laser scanning confocal microscopy was realized using Keyence VK 9710 microscope (laser λ = 408 nm) and a 1D profile of the sample topography was measured along different directions to check its anisotropy.

#### 2.2.2. Polarimetry

Characterization was carried out using a home-built 4-PEM Mueller matrix polarimeter (four photoelastic modulators) [27] in transmission. This spectroscopic instrument was operated in the range from 350 to 850 nm in steps of 2 nm, thus detecting modifications in the polarization of light when a beam propagated through the material by measuring the full Mueller matrix of the sample. The light spot had a diameter of 1.5 mm, and measurements were performed in central parts of the samples. By reducing the raw polarization transfer or Mueller matrices to their differential generators using the analytic inversion method [28], samples were characterized in terms of linear birefringence (LB), linear dichroism (LD), 45° linear birefringence projection (LB’), 45° linear dichroism projection (LD’), circular birefringence (CB), and circular dichroism (CD).

Direction-dependent optical properties along reference axes are usually quantified via LB and LD, thereby accounting for linear anisotropy of a sample. In contrast, optical properties depending on the rotation sense of light are quantified via CB and CD, which account for circular anisotropy of materials. In Table 1, these properties are defined in terms of the real (*n*) parts and the imaginary (*k*) parts, respectively, of the effective refractive index (*n* + *ik*) of the material.

## 3. Results and Discussion

### 3.1. Etching Process

#### 3.1.1. Ion Bombardment Steps

The monolayer of the silica particles served as a 2D colloidal lithography mask, effectively transferring the pattern of the colloidal template onto the crystalline silicon (c-Si) and the glass substrates through ion beam etching at oblique incidence (*θ* = 45°) [22]. Following each etching step, the sample was rotated in its plane at an azimuth angle *ϕ* for the subsequent etching process. The azimuth angle *ϕ* was varied in steps, Δ*ϕ*, of 45°, 60°, 90°, 120°, and 135° in the different samples. Three etching steps were performed on each sample, rotating stepwise by a given Δ*ϕ* value. The duration of the first etch was 45 min. The second and third etching steps were performed for 30 and 15 min, respectively, to minimize the erosion of the most recent features by the subsequent ion beam etchings. Indeed, a side effect during dry etching is the shape modification of the particles due to the comparable sputter yields of the silica mask and silicon substrate [22].

**Figure 2:**
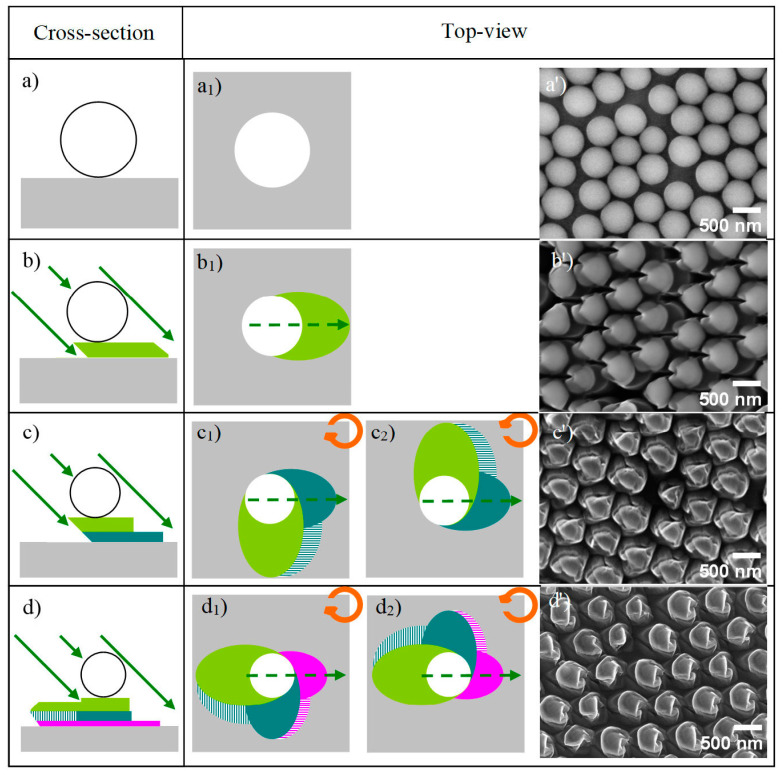
Scheme of the etching process at oblique incidence in cross-section and top views. The solid green arrows represent the direction of the etching ions, and the dashed green arrows are the projections of the etching direction on the sample surface. (**a**,**a_1_**) Pristine surface with a single silica particle (white disc) on a substrate (grey square); (**b**,**b_1_**) 1st pillar (olive green ellipse) formed in the shadow of the etched particle; (**c**,**c_1_**,**c_2_**) 2nd pillars, orthogonal to the 1st (forest green and striped green ellipses) in the shadow of the etched particle and 1st pillar; (**d**,**d_1_**,**d_2_**) 3rd pillar (magenta and striped magenta ellipses) in the shadows of the etched particle and 2nd pillar. SEM images of the resulting samples. (**a’**) Colloidal crystal before etching; (**b’**) patterned silicon substrate after etching for 45 min; (**c’**) 45 and 30 min CCW; (**d’**) 45, 30, and 15 min counterclockwise (CCW) at azimuthal variations of Δ*ϕ* = 90°. Initial size of silica particle: 558 ± 24 nm.

During the etching process, the contribution of the neighboring particles in a colloidal crystal has an influence on the pillar shape. The shadows of the particles interfere with the structure in a manner depending on the interparticle distance and, also, on the orientation of the particle network in the crystal plane with respect to the etching direction. However, for the purpose of this study, a simplified mechanism assuming isolated particles is proposed. The cross-section and the top views of the mechanism of etching for a substrate coated with one particle are depicted in Figure 2, where the three steps needed to obtain a chiral structure with azimuthal variations Δ*ϕ* = ±90° are schematized. The formation of pillars arises from the protective effect of the particle whose shadow is projected on the substrate at oblique incidence during the etching steps. The contributions of the pillar shadows from the previous etching steps are also shown in Figure 2 (stripes), but in this case, due to the lateral dimensions of the projected object (a few nanometers), successive pattern transfers have minor effect on the substrate etching compared to the sphere shadow itself. The etching process consisted of the following three steps:

1. The pillar is formed in the shadow of the etched particle (Figure 2b). Then, the crystal is rotated by ±90° around the azimuthal normal in a clockwise (CW) or counterclockwise (CCW) direction, respectively.
2. Two pillars (principal and secondary), orthogonal to the first, are carved in the shadow of the etched particle and first pillar (Figure 2c_1,_c_2_). The crystal is rotated by another azimuth in its plane in the same sense as in the first rotation.
3. Two other pillars (principal and secondary) are formed, whose directions are aligned with that of the first pillar in the shadows of the etched particle and the second pillar (Figure 2d_1,_d_2_).

#### 3.1.2. Enantiomorphous Structures

The shape of the pillars formed during etching depends on the etching time, the oblique incidence of ions, and the azimuthal angle within the crystal plane. The shadowing effect of the particle decreases with the etching time and the remaining particle size. A balance between the formation and destruction of the pillar [22,29] was achieved by the aforementioned etch times of 45 min for the first step, 30 min for the second step, and 15 min for the third step.

The oblique incidence angle was set to *θ* = 45°, representing a compromise between greater angles, which would minimize colloidal lithography, and angles less than 45°, which would be overly destructive [22]. The initial azimuth angles *ϕ* specified above were evaluated. The projection of the initial etching direction was always set in line with the square samples.

**Figure 3:**
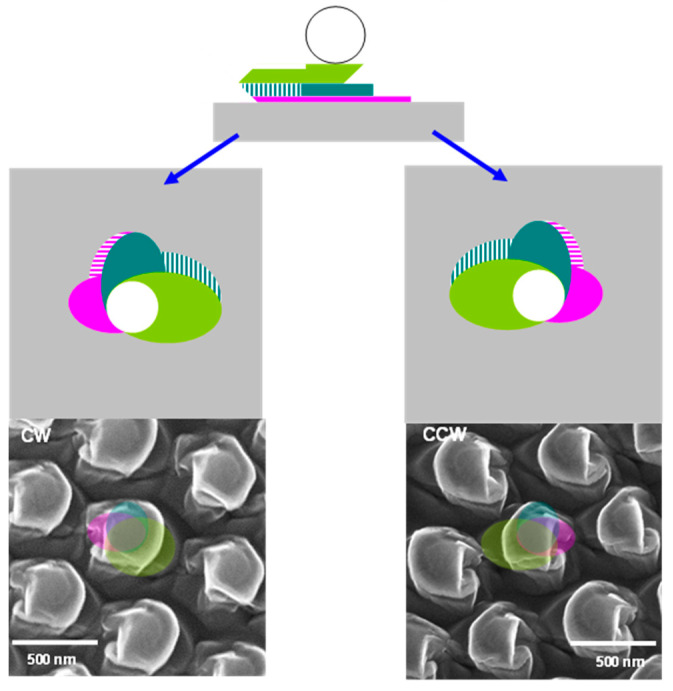
Scheme of the etching and etching results (SEM) for CW (**left**) and CCW (**right**) patterned samples. False colors were added to the SEM images so that they can be compared with the schemes above.

Depending on the sense of the azimuth angle rotation (+/−), structures with opposite handedness (enantiomorphs) were generated (identified as CW/CCW as shown in Figure 3). After the second etching step, the sense of rotation is continued in the third (last) step. In Figure 2, the olive green, forest green, and magenta areas correspond to the pillars carved in the underlying substrate protected by the particle shadow for the progressively shorter etches. Figure 2d_1_,d_2_ correspond to the third step with the same etching duration; the only difference is the change in the azimuthal angle in the CW and CCW samples.

**Figure 4:**
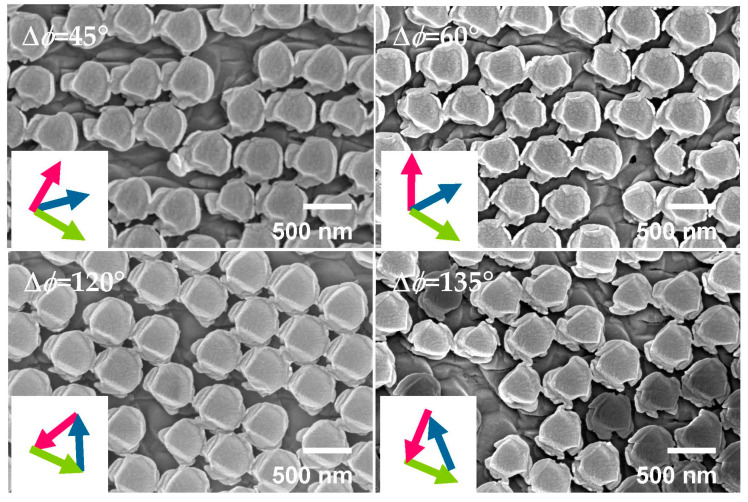
SEM images of samples after complete etching sequences. Different variations of azimuthal angle between etching steps were tested, i.e., Δ*ϕ* = 45°, 60°, 120°, and 135°. Olive green, forest green, and magenta arrows show the etching directions for the 1st, 2nd, and 3rd steps, respectively.

The different azimuth angles *ϕ* between the consecutive etching steps were evaluated. The shape of the pillars and particles change as a function of Δ*ϕ* (Figure 4). The contrast among the particles is likely an artifact due to the difference in the individual particle groundings following etching.

#### 3.1.3. Surface Chemistry

**Figure 5:**
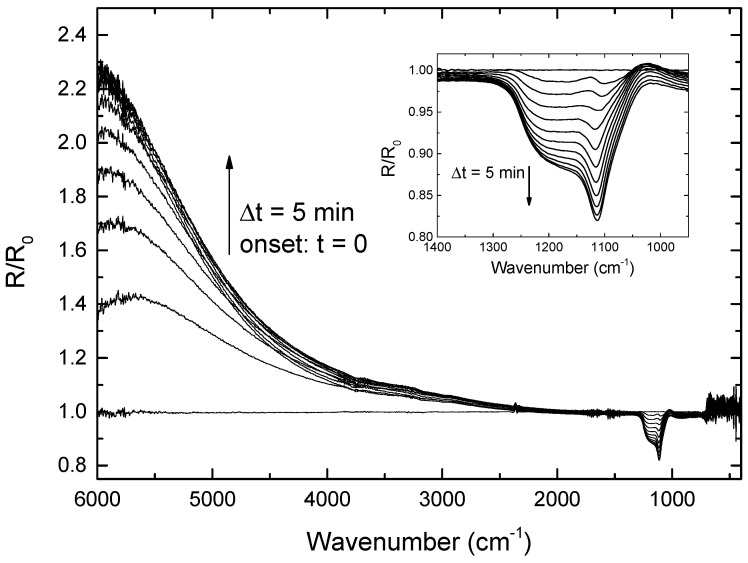
Evolution of the normalized IR spectra, measured in reflection, during etching at normal incidence with Ar^+^ ions at a kinetic energy of 500 eV. Spectra recorded every 5 min. Inset: 950–1400 cm^−1^ region enlarged.

Figure 5 shows the evolution of the normalized IR spectra in reflection mode during Ar^+^ etching at normal incidence (500 eV). Each spectrum, *R*, is divided by the reference measured at the onset of the Ar^+^ bombardment, *R*_0_. The samples consist of a compact monolayer of 600 nm silica particles on a silicon wafer. Two regions can be distinguished. The first region is located at high wavenumbers (2000–6000 cm^−1^), where the enhancement in reflectivity evidences the changes in the surface morphology due to nanoparticle etching. The decrease in roughness is correlated with greater reflectivity at lower wavelengths. Saturation in the reflectance after 35–40 min corresponds to the end point in the sputtering of the nanoparticles at ion fluences of 9.9 × 10^17^ and 11.3 × 10^17^ cm^−2^. A second region is situated at low wavenumbers (900–2000 cm^−1^), for which changes in the chemical composition can be observed (inset). The absorption increases in the region of the spectrum corresponding to interstitial oxygen (around 1100 cm^−1^), indicating ion-induced implantation of oxygen atoms from the silica nanoparticles as verified by the FTIR analysis (Figure 5). The weak increase in the reflectivity at 1030 cm^−1^ arises in a decrease in SiO_2_ bonds [30]. No saturation is observed in this region.

### 3.2. Structure and Topography 

**Figure 6:**
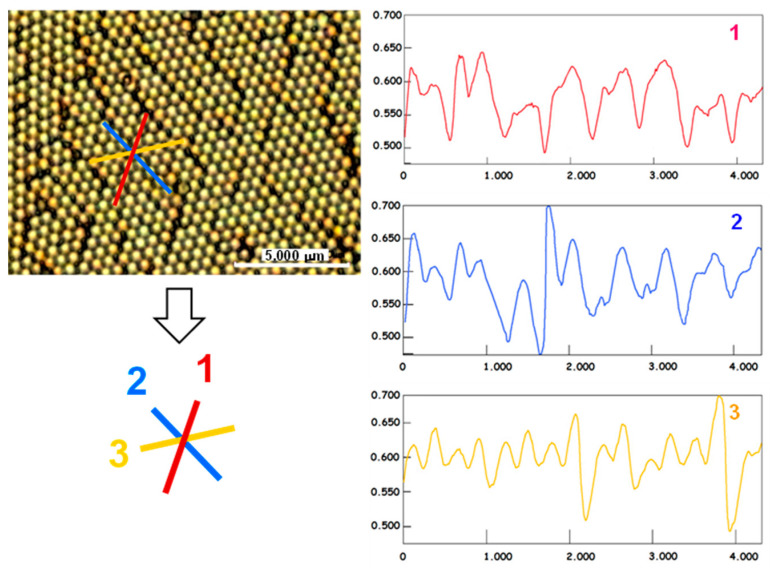
Confocal profilometry along the three mirror line directions 1, 2, and 3 of the original 2D crystal. The axes are measured in microns. Δ*ϕ* = 90°.

Pseudo-two-dimensional hexagonal close-packed particles have three lines of symmetry. The symmetry is not preserved after the three etching steps, as evidenced by the features along lines 1, 2, and 3 on the etched sample in Figure 6, with Δ*ϕ* = ±90°. The profiles along the symmetry axes were probed by the profilometry tool of the confocal microscope. They did not correspond to a perfect 2D hexagonal crystal. This means that, as expected, the structure became anisotropic after the etching steps. The amplitudes and periods vary in the three profiles.

**Figure 7:**
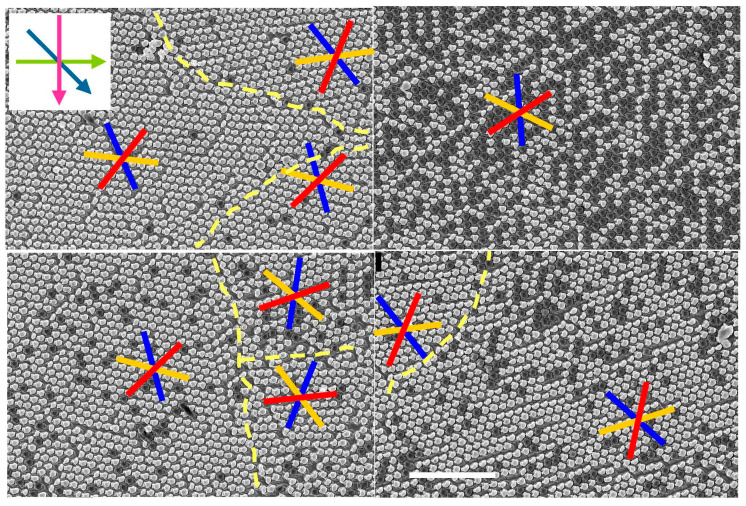
SEM images of a sample etched thrice with etching angles *θ* = 45° and Δ*ϕ* = −45° (olive green, forest green, and magenta arrows in the inset, top left). The domain boundaries are highlighted with dashed yellow lines. The mirror lines (orange, blue, and red) are misoriented in adjacent grains. The white scale bar is 5 μm and applies to all images.

As expected, the crystals have grain boundaries that might affect the perceived anisotropy. The samples analyzed here consist of multi-domain crystals as previously described [31] and are shown in Figure 7. The grain boundaries are indicated by dashed yellow lines and, generally, they enclose crystal domains that are smaller than the beam spot used for optical characterization (see next section). Thus, a polarimetric analysis can only provide average values over the many crystal domain orientations captured by the incident light.

**Figure 8:**
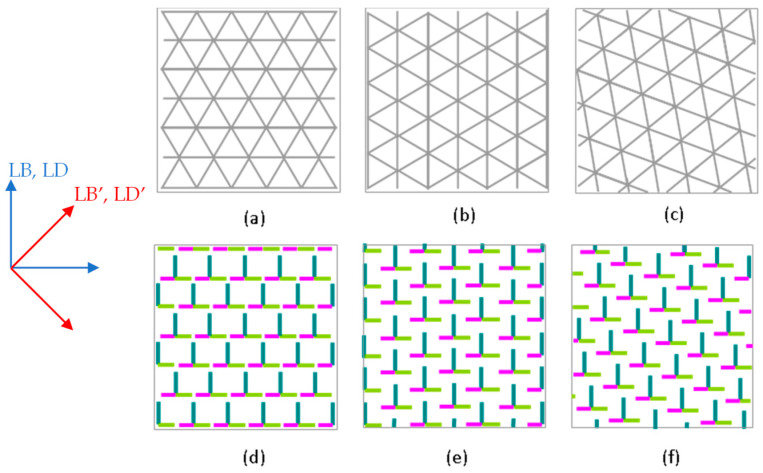
Top view representation of the pillar array structure versus orientations of the colloidal crystal. The etching angle step is set at 90° and the sample substrate is square. The etching directions are taken perpendicular to the sample edges. (**a**–**c**) The grey triangular lattice represents the colloidal crystal structure with different orientations relative to the sample edge. (**b**) is rotated clockwise by 30° and (**c**) is rotated clockwise by 20°. (**d**–**f**) The resulting etched samples are represented on the lower part of the figure. The tri-colored T shapes are the schematized pillars. The blue and red arrows on the left show the directions taken to measure, respectively, LB and LD, and LB’ and LD’.

Due to the template-assisted etching process, in which the colloidal crystal structure is transferred onto the underlying substrate, the parameters of the pillar arrays are dependent on the orientation of the original particle self-assembly. Figure 8 presents a scheme highlighting the difference in the orientation of the crystal domains and the etching direction (Δ*ϕ* = −90°). In this scheme, we can observe the changes in the pillar array structure and their influence on the etched landscape. The crystal domain’s orientation is random, but the etching directions are aligned with the square sample and imparts an overall linear anisotropy.

### 3.3. Optical Properties 

#### 3.3.1. Linear Anisotropy Parameters

In this section, we distinguish between the Au-coated and pristine (uncoated) samples. The uncoated samples show a small linear anisotropy represented by the LB and an apparent LD of about ±0.01–0.02° (spectra not shown here). The anisotropy is expected to be modest due to the multiple domains arising in the Langmuir–Blodgett deposition process [31], but it is optically anisotropic, nonetheless. The origin of the effective dichroic response is more difficult to determine. Here, it is labeled as the apparent LD and may be related to dispersive extinction from the sample (anisotropic scattering, diffraction, etc.) and to undefined optical absorption from the etched silica particles (impurities, defects such as oxygen vacancies due to preferential sputtering, etc.).

**Figure 9:**
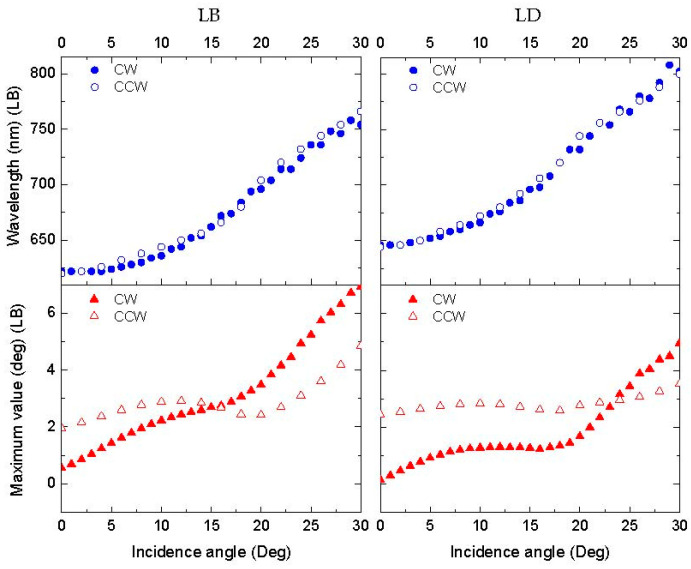
Evolutions of LB and LD peak values and the corresponding wavelengths as a function of the incidence angle of the light in CW and CCW samples. Au coating thickness is 10 nm. Δ*ϕ* = 90°.

In the polarimetric measurements on the Au-coated samples, the angle of incidence of light was varied. A shift in the extrema of the LB and LD to larger wavelengths is observed at higher incidence angles (Figure 9) both for the CW and CCW samples due to the increasing contribution of the component of the wavevector parallel to the plasmon excitation. After coating, a strong enhancement of the linear anisotropy by approximately five-fold was observed. This result is similar to the work of Elliott [32], in which a periodic array of elliptical nanoholes acted as a two-dimensional birefringent crystal, and the optical properties depended on the polarization of the incident light.

**Figure 10:**
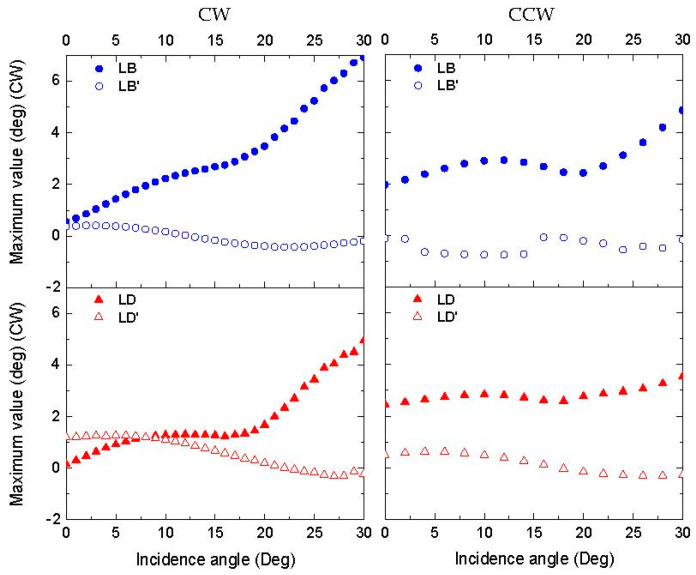
Evolutions of LB, LB’, LD, and LD’ peak values as a function of the incidence angle in CW and CCW samples. Au coating thickness is 10 nm. Δ*ϕ* = 90°.

A characteristic of the polarimetric measurements is that the sides of the sample were aligned with the vertical and horizontal axes of the laboratory reference frame. Due to this arrangement, the anisotropy directions obtained from the measurements of the LB, LB’, LD, and LD’ at small angles of incidence seem to be mostly related to the direction of etching, since it also used the sides of the sample as reference (Figure 10). The anisotropy created via the etching process results in longer pillars in one direction compared to the perpendicular pillars. This is reflected in the relative values of the LB’/LD’ and LB/LD. The anisotropy in the 0° and 90° directions in the surface plane could account for the larger values of the LB and LD compared to the LB’ and LD’, which are shifted 45° (Figure 8). When increasing the incidence angle of the light, the linear anisotropies in Figure 10 are not identical for CW and CCW etching. Symmetry arguments indicate that this arises in slight differences in the pillar structure due to imperfections in sample preparation. Figure 9 shows that the wavelengths of the maxima for the LB (625 to 750 nm) and LD (650 to 800 nm) are almost identical for the CW and CCW samples, indicating that both samples essentially have the same periodicity of the colloidal nanostructure.

#### 3.3.2. Circular Anisotropy Parameters

**Figure 11:**
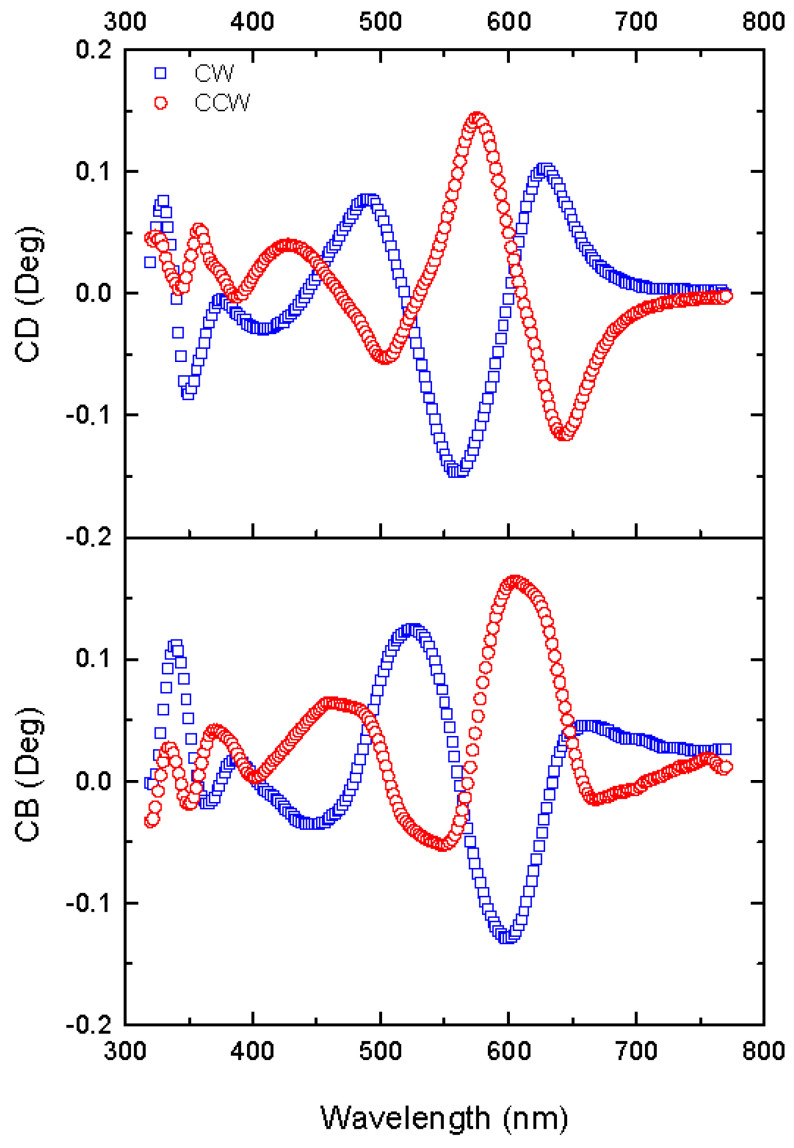
Spectra of CD and CB in uncoated CW and CCW samples. Δ*ϕ* = ±90°.

The uncoated etched samples show optical activity; the CB and CD (circular diattenuation/extinction or apparent circular dichroism) values of about ±0.15° are ten times larger than the values measured for the LB and LD (Figure 11), and their largest values are located between 550 and 650 nm. The response of the CW and CCW samples are close to mirror-symmetric as expected due to the opposite sense of the screw-shaped pillars, but with a small redshift of the CB and CD response in the CCW samples in comparison to the CW samples. As in the case of linear anisotropy, the observed diattenuation, CD, is attributed to optical extinction phenomena related to scattering and residual absorption.

The polarimetric characterization showed that the deposition of a thin gold coating (10 nm) was sufficient to increase the chiroptical response by a factor of five. Such an enhancement accounts for the plasmonic interaction between the incident light and the gold-coated nanostructures [33]. In addition, a further signal enhancement was observed with the increase in the angle of incidence.

**Figure 12:**
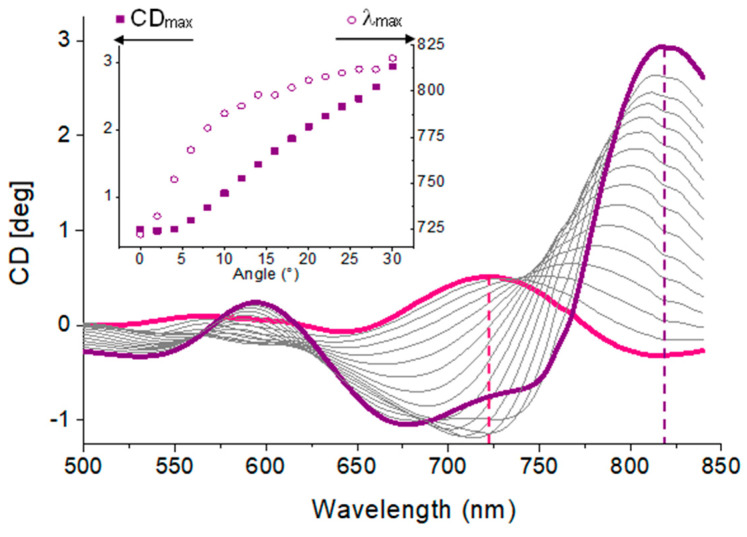
CD spectra in a gold-coated CCW sample as a function of incidence angle from 0° to 30°. Inset: Evolution of numerical CD extrema plotted. Δ*ϕ* = 90°.

Figure 12 shows the CD in a gold-coated sample increasing as a function of the incidence angle due to the parallel component *k*_//_ of the wavevector in plasmon excitation. This is a consequence of the coupling of the electric field and reciprocal lattice vector of the sample. The CB and CD extrema with a value of about 0.75° in the range between 700 and 750 nm at a 0° incidence angle are red shifted to 800 and 850 nm with values of ±3° at a 30° incidence angle.

The stability of the fabricated nanostructures was evidenced by the repeatability of the measured optical parameters. Although the nanostructures were stable, they required isolation from the environment to limit the risk of scratches and dust exposure. An option to increase the lifetime of a device based on this fabrication method can consist of depositing a thin protective layer on top of the lithographed area [29].

## 4. Conclusions

Colloidal lithography is a technique that offers an adequate trade-off between the control of the tailored structure and the size of the lithographed area. The technique presented here requires two main steps after particle synthesis, namely the colloidal crystal self-assembly and the pattern transfer onto the substrate via directional ion beam etching. It is a fast, cost-effective technique, and large patterned areas (mm^2^) can be prepared. The self-assembly step is crucial because the performance of the transferred pattern strongly depends on the quality of the deposited colloidal crystal.

Ion beam etching performed at oblique incidence with different azimuthal angles resulted in the production of a series of nanostructured colloidal samples. By changing the orientation of the azimuthal angle, chiral objects were generated. A three-step etching process was employed to produce chiral gold-coated nanostructures. These optically active samples exhibited maximum CB and CD amplitudes of 0.15° for both etching directions. The optical activity was enhanced by a factor of five after the gold coating. The oblique incidence measurements reveal the role of the in-plane component of the wavevector (*k*_//_) of light in facilitating plasmon couplings between the gold-coated pillars of the nanostructure. Incidence-angle-dependent plasmon couplings were evident in the amplitudes and energies of the CD peaks. The eigendirections of the linear anisotropies (LB and LD) were defined by the sample edge direction with respect to the etching beam.

## Figures and Tables

**Table 1:** Relations between birefringent and dichroic polarimetric properties and optical parameters for a sample with a thickness *l* and an incident wavelength λ. The subscripts (−) and (+) indicate left- and right-circular polarizations, respectively.

Parameter	Definition
Linear birefringence	$LB=\frac{2\pi }{\lambda }\left(n_{x}-n_{y}\right)l$
Linear dichroism	$LD=\frac{2\pi }{\lambda }\left(k_{x}-k_{y}\right)l$
45° LB projection	$LB’=\frac{2\pi }{\lambda }\left(n_{45}-n_{135}\right)l$
45° LD projection	$LD’=\frac{2\pi }{\lambda }\left(k_{45}-k_{135}\right)l$
Circular birefringence	$CB=\frac{2\pi }{\lambda }\left(n_{-}-n_{+}\right)l$
Circular dichroism	$CD=\frac{2\pi }{\lambda }\left(k_{-}-k_{+}\right)l$

## Data Availability

The data presented in this study are available upon request from the corresponding authors.

## References

[1] Chipman R.A., Lam W.-S.T., Young G. (2019). Polarized Light and Optical Systems.

[2] Sacanna S., Pine D.J. (2011). Shape-anisotropic colloids: Building blocks for complex assemblies. Curr. Opin. Coll. Interface Sci..

[3] Zhang W., Potts A., Bagnall D.M., Davidson B.R. (2006). Large area all-dielectric planar chiral metamaterials by electron beam lithography. J. Vac. Sci. Technol. B.

[4] Potts A., Papakostas A., Zheludev N.I., Coles H.J., Greef R., Bagnall D.M. (2003). Planar chiral metamaterials for photonic devices. J. Mater. Sci. Mater. Electron..

[5] Potts A., Papakostas A., Bagnall D.M., Zheludev N.I. (2004). Planar chiral meta-materials for optical applications. Microelectron. Eng..

[6] Lu B.-R., Wan J., Shu Z., Xie S.-Q., Chen Y., Huq E., Qu X.-P., Liu R. (2009). Metallic and dielectric photonic crystals with chiral elements by combined nanoimprint and reversal lithography in SU-8. Microelectron. Eng..

[7] Raub A.K., Brueck S.R.J. (2011). Large area 3D helical photonic crystals. J. Vac. Sci. Technol. B.

[8] Retsch M., Tamm M., Bocchio N., Horn N., Förch R., Jonas U., Kreiter M. (2009). Parallel Preparation of Densely Packed Arrays of 150-nm Gold-Nanocrescent Resonators in Three Dimensions. Small.

[9] Oliveira R.D., Mouquinho A., Centeno P., Alexandre M., Haque S., Martins R., Fortunato E., Aguas H., Mendes M.J. (2021). Colloidal Lithography for Photovoltaics: An Attractive Route for Light Management. Nanomaterials.

[10] Frank B., Yin X., Schäferling M., Zhao J., Hein S.M., Braun P.V., Giessen H. (2013). Large-Area 3D Chiral Plasmonic Structures. ACS Nano.

[11] Ogier R., Fang Y., Svedendahl M., Johansson P., Käll M. (2014). Macroscopic Layers of Chiral Plasmonic Nanoparticle Oligomers from Colloidal Lithography. ACS Photonics.

[12] Shao L., Zheng J. (2019). Fabrication of plasmonic nanostructures by hole-mask colloidal lithography: Recent development. Appl. Mater. Today.

[13] Yang Y.C., Sheu J.-K., Lee M.-L., Yen C.H., Lai W.-C., Hon S.J., Ko T.K. (2012). Vertical InGaN light-emitting diode with a retained patterned sapphire layer. Opt. Express.

[14] Bochenkov V.E., Sutherland D.S. (2018). Chiral plasmonic nanocrescents: Large-area fabrication and optical properties. Opt. Express.

[15] Klös G., Miola M., Sutherland D.S. (2019). Increased Refractive Index Sensitivity by Circular Dichroism Sensing through Reduced Substrate Effect. J. Phys. Chem. C.

[16] Guan Y., Wang Z., Ai B., Chen C., Zhang W., Wang Y., Zhang G. (2020). Chiral Plasmonic Metamaterials with Tunable Chirality. ACS Appl. Mater. Interfaces.

[17] Hentschel M., Schäferling M., Duan X., Giessen H., Liu N. (2017). Chiral plasmonics. Sci. Adv..

[18] Goerlitzer E.S.A., Puri A.S., Moses J.J., Poulikakos L.V., Vogel N. (2021). The Beginner’s Guide to Chiral Plasmonics: Mostly Harmless Theory and the Design of Large-Area Substrates. Adv. Opt. Mater..

[19] Wu W., Pauly M. (2022). Chiral plasmonic nanostructures: Recent advances in their synthesis and applications. Mater. Adv..

[20] Ruffato G., Romanato F. (2012). Grating-coupled surface plasmon resonance in conical mounting with polarization modulation. Opt. Lett..

[21] Hendry E., Carpy T., Johnston J., Popland M., Mikhaylovskiy R.V., Lapthorn A.J., Kelly S.M., Barron L.D., Gadegaard N., Kadodwala M. (2010). Ultrasensitive detection and characterization of biomolecules using superchiral fields. Nat. Nanotechnol..

[22] Portal S., Rubio-Roy M., Corbella C., Vallvé M.A., Ignes-Mullol J., Bertran E. (2009). Influence of incident ion beam angle on dry etching of silica sub-micron particles deposited on Si substrates. Thin Solid Films.

[23] Stöber W., Fink A., Bohn E. (1968). Controlled growth of monodisperse silica spheres in the micron size range. J. Colloid Interface Sci..

[24] Portal S., Vallvé M.A., Arteaga O., Ignés-Mullol J., Canillas A., Bertran E. (2008). Optical characterization of colloidal crystals based on dissymmetric metal-coated oxide submicrospheres. Thin Solid Films.

[25] Takeuchi T., Corbella C., Grosse-Kreul S., von Keudell A., Ishikawa K., Kondo H., Takeda K., Sekine M., Hori M. (2013). Development of the sputtering yields of ArF photoresist after the onset of argon ion bombardment. J. Appl. Phys..

[26] Corbella C., Grosse-Kreul S., Kreiter O., de los Arcos T., Benedikt J., von Keudell A. (2013). Particle beam experiments for the analysis of reactive sputtering processes in metals and polymer surfaces. Rev. Sci. Instrum..

[27] Arteaga O., Freudenthal J., Wang B., Kahr B. (2012). Mueller matrix polarimetry with four photoelastic modulators: Theory and calibration. Appl. Opt..

[28] Arteaga O., Canillas A. (2010). Analytic inversion of the Mueller-Jones polarization matrices for homogeneous media: Erratum. Opt. Lett..

[29] Corbella C., Portal-Marco S., Rubio-Roy M., Bertran E., Oncins G., Vallvé M.A., Ignés-Mullol J., Andújar J.L. (2011). Modifying surface properties of diamond-like carbon films via nanotexturing. J. Phys. D Appl. Phys..

[30] Kajiyama K., Yoneda T., Fujioka Y., Kido Y. (1997). Si-O bond formation by oxygen implantation into silicon. Nucl. Instrum. Meth. Phys. Res. B.

[31] Portal-Marco S., Vallvé M.A., Arteaga O., Ignés-Mullol J., Corbella C., Bertran E. (2012). Structure and physical properties of colloidal crystals made of silica particles. Colloids Surf. A Physicochem. Eng. Asp..

[32] Elliott J., Smolyaninov I.I., Zheludev N.I., Zayats A.V. (2004). Wavelength dependent birefringence of surface plasmon polaritonic crystals. Phys. Rev. B.

[33] Eftekhari F., Davis T.J. (2012). Strong chiral optical response from planar arrays of subwavelength metallic structures supporting surface plasmon resonances. Phys. Rev. B.

